# Myxoid Epithelial Leiomyoma of the Vulva: A Case Report and Literature Review

**DOI:** 10.1155/2015/894830

**Published:** 2015-06-22

**Authors:** Ting Zhao, Xishi Liu, Yuan Lu

**Affiliations:** Obstetrics and Gynecology Hospital, Fudan University, Shanghai 200011, China

## Abstract

Leiomyoma of vulva is rare, and usually misdiagnosed clinically as Bartholin cyst. It usually presents spindle-shaped tumor cells, but some rare cases consisted mainly of atypical epithelioid tumor cells. We report here a case of 30-year-old woman consulting with a vulvar mass of 7 cm in the Bartholin glands area. The lesion was surgically excised with its capsule completely. Pathological examination and immunochemistry showed characteristic of epithelioid leiomyoma with myxoid stroma with both estrogen receptor (ER) and progesterone receptor (PR) staining negative, which was really rare as only 2 cases of vulvar leiomyoma with both ER and PR were reported before.

## 1. Background

Smooth muscle tumors of the vulva are rare and are usually misdiagnosed clinically as Bartholin cysts. These tumors are considered to originate from smooth muscle within erectile tissue, blood vessel walls, the round ligament, the dartos muscle, or the arrector pili muscle [1] and from the stem cells localized in Bartholin's gland [2]. Typical vulvar smooth muscle tumors demonstrated spindle-shaped cells, but other histological types such as epithelioid tumors are also reported. As a rare variant, epithelioid leiomyoma often presents plump, round cells with abundant eosinophilic and vacuolated-to-clear cytoplasm [3]. Complex morphological features of smooth muscle tumors of the vulva usually give rise to diagnostic difficulties. As a result, immunohistochemistry plays an important role in differential diagnoses. These tumors usually present positive for anti-smooth muscle antibody (SMA), desmin, and vimentin. ER and PR are positive in some but not all the cases. Here we report a case of vulvar epithelioid leiomyoma which was misdiagnosed with Bartholin cysts and confused us for the untypical microscopical findings and immunohistochemistry results.

## 2. Report of a Case

A 30-year-old Chinese G2P1 female had found a mass in her left labium major for more than 7 years. The mass was about 1 cm when first discovered and asymptomatic in the next 6 years and then showed obvious enlargement in the last year. The patient did not feel any discomfort and never went to hospital until this visit. Her general physical examination was normal except for a palpable soft mass of about 7 cm involving the lower half of left labium major. During the 7 years the patient had a history of full-term pregnancy of cesarean delivery and an induced abortion of the first trimester. The family and patient's past medical history were not significant.

The mass was considered clinically to be a Bartholin gland cyst before surgery. When mucous between hymen and left labium minor was incised, soft fragile tissue of raw fish appearance was found but well circumscribed with a capsule. The mass with its wall was carefully excised. The patient had a good postoperative recovery with no complications. Recurrence was not found in the next 14 months.

## 3. Pathological Findings

**Figure 1:**
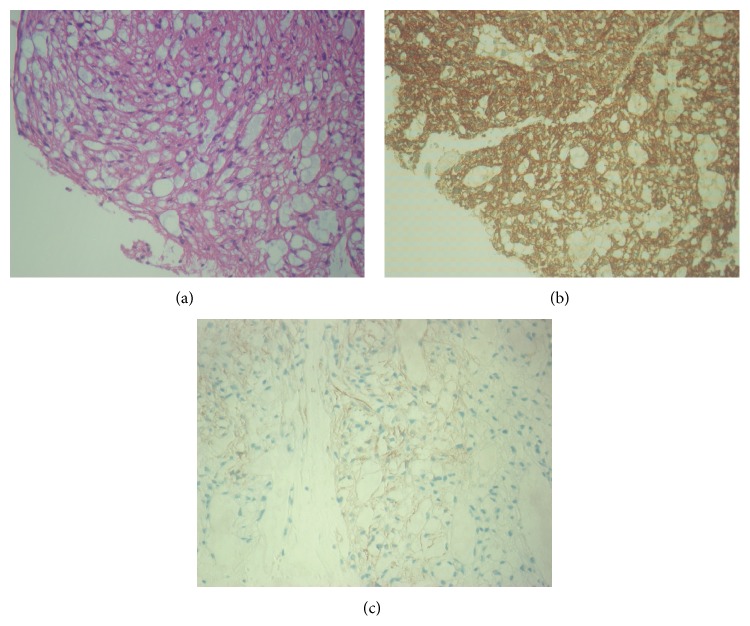
The microscopical features of the neoplasm. (a) Hematoxylin and eosin staining. (b) The immunohistochemistry staining of smooth muscle actin in the neoplasm. (c) The immunohistochemistry staining of desmin in the neoplasm.

The gross specimen consisted of a soft mass measuring 7 cm in diameter. The cut surfaces were pink to white gray in color with a rubbery and fleshy quality. Microscopically, the neoplasm consisted of epithelioid cells arranged in nests filled with mucous material. The nuclear morphology was uniform throughout and consisted of round, regularly shaped nuclei with a single prominent nucleolus. Mitotic figure was rare, and no atypical mitoses were found. The tumor was sharply demarcated peripherally and was covered throughout by a thin layer of delicate fibrous tissue. The immunohistochemical staining pattern of our case, strong positivity for SMA and caldesmon, and focal positivity for desmin (Figure 1) are indicative of smooth muscle differentiation. The tumor was negative for AE1/AE3 (pancytokeratin) and CD31 which tends to exclude epithelium and endothelium differentiation [4]. S-100, CD56, and HMB45 were also negative, which can help to exclude neural originated tumor and melanoma. ER and PR were both negative. The final pathologic diagnosis was a vulvar leiomyoma.

## 4. Discussion

Vulvar leiomyoma is rare. Reidel found only one leiomyoma after reviewing 144 vulvar tumors [5]. There are several histological types of vulvar leiomyoma. Nielsen et al. reviewed the pathological features of 25 cases of vulvar smooth muscle tumor and found 14 tumors consisted mainly of spindle cells arranged in fascicles and 7 cases consisted predominantly of epithelioid neoplastic cells of eosinophilic cytoplasm. The rest 4 tumors presented equal number of spindle and epithelioid cells. Even the 7 epithelioid cases can be divided into two subgroups; one presented cells arranged in anastomosing cords and often encircled with blood vessel walls; the other presented cells arranged in nests [6]. In other reports, the same multimorphology was found but the epithelioid type is much less as Tavassoli found only 1 out of 32 and Newman found 2 out of 18 cases of vulvar smooth muscle tumors [3, 7].

As a rare variant, only a few cases of vulvar epithelioid leiomyoma have been reported in the English literature. We reviewed the literature in PubMed and summarized the reports of vulvar epithelioid leiomyoma in Table 1. The reported age varied from 17 to 45 years and the tumor size ranged from 1.5 cm to more than 10 cm.

For our case, we diagnosed the mass as Bartholin gland cyst for the first impression. After surgery we encountered difficulty in making a definite diagnosis as the myxoid appearance was extremely rare. Supportive immunohistochemistry is important for diagnosis and differentiation besides hematoxylin and eosin-stained sides when neoplasms arise at unusual sites. We examined several indicators and found strong positivity for SMA and caldesmon and focal positivity for desmin, which is indicative of smooth muscle differentiation. ER and PR staining were negative which was quite different from leiomyoma from uterine.

It was suggested that vulvar leiomyoma was an estrogen dependent neoplasm [8, 9]. Estrogen-progesterone therapy was closely related to recurrent vulval leiomyoma [8]. A case of vulvar leiomyoma was reported to be finally cured with estrogen receptor modulator following three consecutive excision procedures [9]. But in our case both ER and PR staining were negative. We reviewed the literature and found that only two cases of vulvar leiomyoma with both ER and PR negative had been reported before in the literature [6, 10].

ER and PR are highly expressed in uterine leiomyoma. It can be interpreted that leiomyoma originates from ER/PR expressing myometrial smooth muscle cells. But smooth muscle tumors of vulva were thought to originate from smooth muscle within erectile tissue or blood vessel walls, the round ligament, the dartos muscle, or the arrector pili muscle, which may lack intrinsic ER/PR expression. As Malcolm reported, there was a significant decline of ER staining in epidermis and fibroblast from vagina towards labia minora, labia majora, and the suprapubic skin. In their report ER staining was not seen in skin appendages, blood vessels, and lymphatics except for occasional sweat glands; for PR, no staining was seen in vulvar skin appendages or vessels [11].

The myxoid material of epithelioid leiomyoma of vulva can be digested by hyaluronic acid, indicating that it was composed of hyaluronic acid [3]. The myxoid change was speculated to be degenerative phenomenon related to hormonal changes during the pregnancy [12]. But it is quite obscure if the myxoid tumor presents both ER and PR negative. In Nielsen's series 2 patients were pregnant when the diagnosis was made; one was ER and PR negative while the other was positive. The positive case showed prominent myxoid change while the negative one did not. In Tavassoli's series 7 patients were pregnant when the diagnosis was made and all of them showed noticeably enlargement during gestation and presented prominent myxoid change. Myxoid change was reported in two other cases of pregnant patients; one is ER and PR negative; the other showed weak ER and PR positivity in local areas [4, 10]. For our case, the patient was not pregnant when diagnosed but had gone through a full-term pregnancy of cesarean delivery and an induced abortion of the first trimester during the course of disease. But as the ER and PR were negative, the relationship between pregnancy and myxoid morphology warrants further study.

The most confusing problem with vulvar smooth muscle tumor is to distinguish benign from malignant forms. There are significant differences between the criteria used for smooth muscle tumors of uterine and soft tissues. Significant mitotic activity, focal degenerative cellular atypia, and/or hyaline necrosis can be present in benign uterine leiomyoma, which may be thought of as features of leiomyosarcoma of other places [13]. Tavassoli presumed that >5 cm in diameter, infiltrative margins, and >=5 mitotic figures per 10 HPF were features likely to predict recurrence; vulvar smooth muscle tumors have infiltrative margins (with or without other features), or fulfilling the other two features should be considered as leiomyosarcoma. But the sample was too limited as only 4 recurring out of 32 cases [3]. Nielsen et al. concluded the features of recurrent and metastatic cases and modified that moderate to severe cytological atypia was another feature besides the above three ones. He proposed that tumors manifest three or all of the four features which should be considered sarcomas. If two characteristics were fulfilled the tumor should be classified as benign and atypical leiomyoma; if one or none was fulfilled, then it should be leiomyoma [6]. According to his perspective our case was 7 cm in greatest dimension which fits only one of the criteria and should be considered to be leiomyoma. When the morphological diagnosis is difficult, biomarkers can also be used to predict the behavior of tumor such as p16, p53, and ki-67 [13].

Local excision of tumor with its capsule and a surrounding rim of normal tissue was recommended for the patient [6]. In Nielsen's series most leiomyoma patients did not show recurrence after 2 years from excision, and one patient showed recurrence after 10 years. It was also reported that 11 years after the initial excision a 67-year-old woman presented recurred leiomyoma [14]. Long time follow-up is emphasized.

## Figures and Tables

**Table 1:** A summary of the epithelioid leiomyomas of the vulva.

Author, year	Number	Age	Size (cm)	Myxoid	Outcome
Tavassoli and Norris, 1979 [3]	1	25	1.5	No	Recurrence after 11 mo

Chen et al., 1980 [15]	2	43	8	Yes	Recurrence in 1 yr and died 22 mo after diagnosis

Aneiros et al., 1982 [16]	3	26	—	—	—

Newman and Fletcher, 1991 [7]	4	—	—	—	—
	5	—	—	—	—

Nielsen et al., 1996 [6]	6	28	3	Yes	No recurrence in 3 yr and 5 mo
	7	47	1.6	No	No recurrence in 2 yr
	8	19	4	Yes	No recurrence in 15 mo
	9	20	3	Yes	Recurrence after 10 yr
	10	45	4	No	No recurrence in 5 mo
	11	24	—	No	—
	12	17	5	Yes	—

Hopkins-Luna et al., 1999 [17]	13	45	10.5 × 9.0 × 5.2	Yes	—

Kajiwara et al., 2002 [10]	14	29	4 × 4 × 4.5	Yes	—

Zhou et al., 2006 [4]	15	29	8.5 × 7.5 × 6.5	Yes	No recurrence in 29 mo

Our case, 2013	16	30	7	Yes	No recurrence in 14 mo
